# Directed screening and spatial coupling of farnesyl diphosphate synthase for enhancing menaquinone-7 production in *Bacillus subtilis*

**DOI:** 10.1186/s12934-026-02930-1

**Published:** 2026-01-14

**Authors:** Xiumin Ding, Rui Zhang, Ying Liu, Liang Hong, Yalan Feng, Qiang Li, Zhiming Zheng, Genhai Zhao

**Affiliations:** 1 School of Laboratory Medicine, Wannan Medical College, Wuhu, 241002 China; 2 Institute of Intelligent Machines, Hefei Institutes of Physical Science, Chinese Academy of Sciences, Hefei, 230031 China; 3 School of Life Sciences, Anhui Medical University, Hefei, 230012 China; 4 School of Life Sciences, Wuchang University of Technology, Wuhan, 430223 China

**Keywords:** Farnesyl diphosphate synthase, Directed screening, Spatial coupling, *Bacillus subtilis*, Menaquinone-7

## Abstract

**Background:**

Menaquinone-7 (MK-7), a highly bioactive form of vitamin K₂ with a long half-life, plays pivotal roles in preventing osteoporosis and cardiovascular diseases. A metabolically balanced MK-7-producing strain, *Bacillus subtilis* BS016, has been engineered. However, its biosynthetic efficiency remains hindered by bottlenecks, such as low isoprenoid side-chain elongation efficiency.

**Results:**

To address this limitation, a thermophilic farnesyl diphosphate (FPP) synthase from *Geobacillus stearothermophilus*, characterized by high activity and stability, was identified via database mining. This enzyme was modularly assembled with the endogenous *hepS*-*menG*-*hepT* operon in *B. subtilis* BS016, driven by the strong promoter P_hbs_. A synergistic expression cassette was constructed to achieve spatial co-localization of FPP synthesis with downstream isoprenoid side-chain elongation. The resulting engineered strain BS018 achieved a flask yield of 91.1 mg/L, representing an 11% increase, and maintained a stable titer of 87.9 mg/L in a 5-L fermenter. Further optimisation of fermentation conditions (liquid loading, 50 mL/500 mL; initial pH, 7.0; inoculum dose, 8%) ultimately elevated a flask yield to 109.6 mg/L.

**Conclusion:**

This study demonstrates that the directed screening of high activity heterologous enzymes, combined with the spatial co-assembly of endogenous pathways, can effectively reconstruct and enhance metabolic flux. This integrated strategy, combining directed enzyme screening with spatial pathway assembly, provides a generalizable framework for constructing efficient cell factories not only for vitamin K₂ but also for other high-value terpenoids.

**Supplementary Information:**

The online version contains supplementary material available at 10.1186/s12934-026-02930-1.

## Background

Menaquinone-7 (MK-7), a form of the vitamin K₂ family (terpenoidquinones), is characterized by a naphthoquinone ring with a seven-isoprenoid side chain at the C-3 position. MK-7 is distinguished by its longer half-life and higher bioactivity in the human body, which contribute to its role in preventing and treating diseases, such as osteoporosis, cardiovascular diseases, and Alzheimer’s [1–4].

**Fig. 1:**
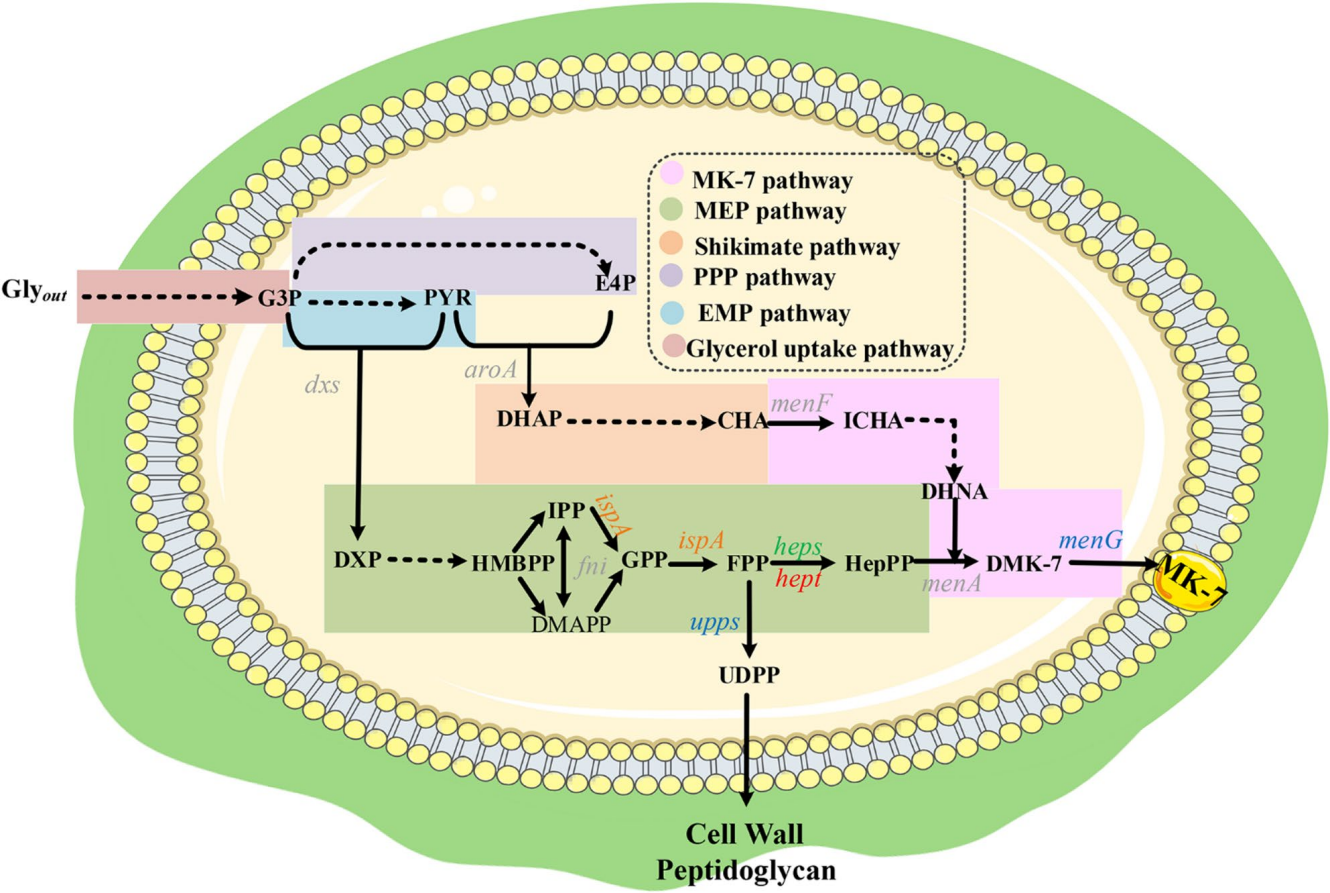
Menaquinone-7 (MK-7) biosynthetic pathway in *B. subtilis* BS016. The biosynthetic pathway of MK-7 in *B. subtilis* primarily comprises seven modules: the glycerol assimilation, MK-7, shikimate (SA), methylerythritol phosphate (MEP), pentose phosphate (PPP), and glycolysis (EMP) pathways, as well as the tricarboxylic acid (TCA) cycle. Compounds involved in the reactions are labeled in black text, and genes that were previously enhanced through overexpression are labeled in gray text. The artwork used in this figure was adapted from Servier Medical Art (http://smart.servier.com/). Servier Medical Art is licensed under Creative Commons Attribution 3.0 Unported License

Consequently, microbial fermentation-based MK-7 biosynthesis has garnered attention due to its eco-friendly nature and cost advantages. However, the biosynthetic pathway of MK-7 is highly complex, and the inherent cytotoxicity of high MK-7 concentrations further compromises fermentation yields (Fig. 1) [5–10]. Although synthetic biology strategies, such as the “push-pull-block” approach, have been used for enhancing metabolic flux, the efficacy of such strategies remains limited [11–14]. In our previous study, enhancement of of key enzymes involved in MK-7 biosynthesis and reconstruction of a cofactor regeneration system in *B. subtilis *168 was achieved. Nevertheless, the flux through the side-chain synthesis module remained inadequate relative to the enhanced upstream supply, leading to local metabolic bottlenecks and insufficient yield improvement [15]. In *B. subtilis*, the efficiency of MK-7 side-chain synthesis primarily depends on the metabolic flux of farnesyl pyrophosphate (FPP), catalyzed by FPP synthase (IspA) in the methylerythritol phosphate (MEP) pathway [16]. FPP is first converted into heptaprenyl diphosphate (HepPP) by the heptaprenyl diphosphate synthase component I/II(HepS/HepT), then HepPP are sequentially transformed into demethylmenaquinone-7 (DMK-7) and MK-7 via 1,4-dihydroxy-2-naphthoate heptaprenyltransferase (MenA) and demethylmenaquinone methyltransferase (MenG), respectively (Fig. 1). However, FPP is also utilized in the synthesis of undecaprenyl diphosphate (UDPP) via undecaprenyl diphosphate synthase (UPPs) (Fig. 1). UDPP is a critical precursor of cell wall biosynthesis [17]. Notably, terpenoid synthases (such as IspA) are often rate-limiting due to their inherently low catalytic efficiency [18, 19]. Therefore, synergistically enhancing IspA enzymatic activity and establishing a directed transport route for FPP toward HepS/HepT are key strategies to overcome the universal bottleneck in the biosynthesis of MK-7.

A key strategy to overcome pathway bottlenecks is the construction of “metabolic channels” in metabolic engineering. This approach involves coupling sequential enzymatic reactions through physical or functional associations. By directing the flow of intermediates, it prevents their diffusion into the cytoplasm or utilization by competing pathways. The core of this strategy lies in achieving spatial co-localization and synergy of key enzymes. Cheah et al. [18] introduced genes encoding nerolidol synthase and farnesyl diphosphate synthase into *Saccharomyces cerevisiae*, increasing nerolidol production from 29.6 mg/L to 4.2 g/L. Similarly, Wang et al. [20] co-expressed FPP and α-farnesene synthases through translational fusion, minimizing loss of FPP to competing pathways and significantly enhancing α-farnesene production. Chen et al. [21] engineered *Yarrowia lipolytica* to produce sclareol through the fusion of (13E)-8α-hydroxylabden-15-yl diphosphate synthase and sclareol synthase. These studies demonstrate the decisive role of spatial proximity in driving metabolic flux. Inspired by this, we propose using operon reconstruction and co-expression strategies to achieve spatial coupling of the MK-7 side-chain synthesis enzyme system in *B. subtilis*, with the goal of alleviating this metabolic bottleneck.

In this study, a highly active and thermostable heterologous GsIspA was identified through database mining. This enzyme was then modularly assembled with the *hepS*-*menG*-*hepT* operon and spatially co-expressed under a strong promoter to construct an efficient metabolic channel. Finally, fermentation process optimization was carried out to further enhance production performance. The strategy of combining enzymes spatial co-localization with pathway enhancement also offers valuable insights for the metabolic engineering of other terpenoid compounds.

## Materials and methods

### Chemicals and reagents

High-performance liquid chromatography (HPLC)-grade organic solvents were purchased from Sigma-Aldrich (Steinheim, Germany). MK-7 standard (≥ 99%) was procured from Fujifilm Wako Pure Chemical Corporation (Japan). The bacterial genome extraction kit was purchased from TIANGEN Biotech (Beijing, China). Plasmid miniprep kits and other chemical reagents were procured from Sangon Biotech Co., Ltd. (Shanghai, China). High-fidelity DNA polymerase (PrimeSTAR HS DNA) was purchased from Takara Bio Inc. (Dalian, China). MonScript™ 5×RTIII All-in-One Mix was purchased from Monad Biotech Co., Ltd (Suzhou, China).

### Bioinformatics tools

The SWISS-MODEL online system (https://swissmodel.expasy.org) was used to obtain the 3D structural model of BsIspA [22, 23]. The VERIFY_3D Server (http://services.mbi.ucla.edu/Verify_3D/) was employed to check the quality of the generated model. Uniport (https://www.uniprot.org/) were utilized to analyze the classes, domains, and characteristic amino acid positions of the protein superfamily to which BsIspA belongs [24]. The amino acid sequences of *G. stearothermophilus* farnesyl diphosphate synthase GsIspA (RefSeq ID: WP_033016440.1) and *B. subtilis* farnesyl diphosphate synthase BsIspA (RefSeq ID: NP_390308.2) were retrieved from the NCBI in FASTA format. Further, the PDB structure of GsIspA (ID: 5AYP) was downloaded. Sequence alignment was performed using the MUSCLE algorithm in MEGA (version 11); the alignment results were exported in the FASTA format. The results of sequence alignment were integrated with the PDB structure using ESPript 3.0 to perform spatial coloring analysis based on site conservation.

### Strains, culture conditions, and growth curve determination

The strains and plasmids used in the present study are listed in Supplementary Material 1 (Table S1). All genetically modified strains were cultured in Luria–Bertani (LB) medium at 37 °C with shaking (220 rpm). *B. subtilis* was inoculated into LB medium and cultured at 37 °C with shaking (220 rpm) for 12 h to prepare the seed culture. Subsequently, the seed culture was transferred at a 10% (v/v) inoculation volume into 500 mL shake flasks containing 50 mL fermentation medium (composition: glycerol, 30 g/L; soybean peptone, 60 g/L; yeast extract, 5 g/L; K₂HPO₄, 3 g/L and MgSO₄·7 H₂O, 0.5 g/L ). The fermentation system was then incubated at 40 °C with shaking (250 rpm) for 120 h. All experiments were performed with three independent biological replicates unless otherwise specified. During this period, 1 mL fermentation broth was collected every 12 h, and thereafter, the optical density was measured at 600 nm (OD₆₀₀) to monitor cell density. All gene editing experiments were based on antibiotic selection strategies, using kanamycin (50 µg/mL), ampicillin (100 µg/mL), and chloramphenicol (5 µg/mL).

### DNA assembly and *B. subtilis* transformation

Multi-fragment DNA assembly was performed by overlap extension using polymerase chain reaction (PCR) [11, 25]. The assembled DNA was sequenced by Sangon Biotech Co., Ltd. (Shanghai, China) and subsequently transformed into competent *B. subtilis* using Spizizen transformation [15, 26]. Primers used for gene modification are listed in Additional File 1 (Table S2).

### Genomic integration of heterogeneous genes

Recombinant strains with integrated heterogeneous genes were constructed as reported previously [11, 25]. First, the nucleotide sequence of *GsispA* from *G. stearothermophilus* was synthesized by Sangon Biotech (Additional File 1: Table S3). Next, the *GsispA* fragment was amplified via PCR with primers gsispa3-F/gsispa3-R. Using the genome of *B. subtilis* BS016 as a template, the upstream homologous arm (heps-up, chrX: 2385410–2384456) was amplified with primers *heps*1-F/*heps*1-R. The downstream homologous arm (*heps*-down, chrX: 238457–2383461) was amplified with primers gsispa4-F/heps4-R. Using plasmid p7C6P_hbs_ as a template, a functional module comprising the lox71-cmR-lox66 cassettes and promoter P_hbs_ was amplified with primers heps2-F/gsispa2-R. Subsequently, four fragments (*heps*-up, lox71-cmR-lox66-P_hbs_, *GsispA*, and *heps*-down) with a total length of 4341 kb were ligated using triple-fusion PCR. The fusion fragment was purified and transfected into competent cells (BS016) using the Spizizen transformation. The resistance marker of the host strain was removed using the Cre/loxp system. The pDGC plasmid was transformed into cmR clones to promote lox71 and lox66 recombination by excising the resistance cassette. Finally, the intracellular plasmid pDGC was eliminated by incubation at 50 °C for 12 h [11]. The method used for overexpressing *BsispA* in BS016 was similar to that used for overexpressing *GsispA*.

### Quantitative RT-PCR analysis

Total RNA of *B. subtilis* was extracted using the RNA-easy Isolation Reagent (Vazyme, Nanjing, China) and reverse-transcribed into cDNA using MonScript™ 5 × RTIII All-in-One Mix (Monad, Suzhou, China) following the manufacturer’s protocol. qRT-PCR was carried out on a LightCycler^®^ 480 system (Roche Diagnostics GmbH, Mannheim, Germany) using MonAmp™Fast SYBR^®^ Green qPCR Mix (Monad, Suzhou, China). The *hbs* was used as an internal reference to assess relative gene expression. Data analysis followed the 2^−ΔΔCt^ method, with reaction conditions based on a previously reported protocol [15].

### Validation of MK-7 fermentation

A three-stage scale-up strategy was used for assessing stability of the *B. subtilis* MK-7 fermentation process in a 5-L bioreactor. In the first stage, a single colony was cultured in 50 mL LB medium at 37 °C with shaking (220 rpm) for 12 h to prepare a primary seed culture. The second stage involved transferring 10% (v/v) of the primary seed into 300 mL fermentation medium and maintaining identical conditions to obtain a secondary seed culture. During fermentation, 300 mL secondary seed (10% inoculation ratio) was added to 3 L fermentation medium in a bioreactor with a liquid loading of 66%. The temperature was set at 40 °C, with the initial dissolved oxygen (DO) at 100%. Agitation began at 400 rpm and was automatically adjusted (400–800 rpm) to maintain the DO at 30%, while aeration was fixed at 2.0 vvm. The initial pH of the medium was set at 7.3, and no further adjustments were made during fermentation. The process lasted for 120 h, and samples were collected every 12 h to monitor bacterial growth (OD_600_) and MK-7 yield. Due to equipment limitations, a single biological replicate was used, but the production of MK-7 was determined in three technical replicates. This allowed dynamic evaluation of the relationship between cell growth and MK-7 synthesis.

### Fermentation condition optimization and MK-7 yield

To investigate the impact of fermentation condition optimization on MK-7 production in *B. subtilis* BS018, three structured experiments were performed to evaluate the key parameters. In the liquid loading optimization experiment, six different fermentation volumes (50 mL, 60 mL, 70 mL, 80 mL, 90 mL, and 100 mL) were tested in 500 mL Erlenmeyer flasks, with the liquid fill ratios ranging between 10 and 20% (v/v). In the initial pH regulation experiment, the pH of the fermentation medium was precisely adjusted to six levels (6.0, 6.5, 7.0, 7.5, 8.0, and 8.5) using 1 M NaOH or HCl prior to autoclaving. In the inoculum optimization experiment, seed culture inoculation ratios of 5, 8, 10, 12, and 15% (v/v) were tested. All experimental groups had three independent biological replicates to ensure reproducibility. MK-7 production was quantified by HPLC. Based on the experimental results, the optimal combination of fermentation parameters was determined to maximize MK-7 yield.

### MK-7 extraction and quantification

To extract MK-7 from the fermentation supernatant, 2 mL bacterial fermentation broth was centrifuged at 9000×*g* for 6 min. After collecting 1 mL supernatant, 2 mL of a hexane: isopropanol mixture (2:1, v/v) was added, followed by vigorous vortexing for 20 min. Subsequently, 1 mL n-butanol was added and vortexing was repeated for another 20 min before centrifugation at 5000×*g* for 3 min. The organic phase was filtered through a 0.22 μm organic filter membrane to obtain the MK-7 extract. With respect to intracellular MK-7 extraction, the bacterial pellet obtained by centrifugation was frozen at − 80 °C and subjected to vacuum freeze-drying. The dried cells were resuspended in 2 mL ethanol, extracted using ultrasonic assistance for 10 min, and vortexed for 20 min. After centrifugation at 5000×*g* for 3 min, the organic phase was collected and filtered through a 0.22 μm filter membrane. The total yield of MK-7 in the fermentation broth was defined as the sum of the MK-7 concentrations in the fermentation supernatant and the bacterial pellet.

MK-7 quantification was performed using an HPLC equipped with an ultraviolet (UV) detector (SPD-16 model) and a reverse-phase C-18 column (VP-ODS, 4.6 mm×250 mm). The mobile phase consisted of a methanol/dichloromethane mixture (4:1, v/v), with a flow rate of 1 mL/min and a column temperature of 35 °C. Detection was conducted at a wavelength of 248 nm, with an injection volume of 20 µL. The method exhibited a linear detection range of 10 to 150 mg/L, with a strong linear correlation (R² = 0.999) between MK-7 concentration and peak area, ensuring high accuracy for quantitative analysis.

## Results and discussion

### FPP synthase screening and analysis

IspA (EC 2.5.1.10) is one of the prenyltransferases and occupies a central branch point in the prenyl chain elongation pathway [27]. In *B. subtilis*, the naturally active of BsIspA exhibits notably low catalytic efficiency, with a specific activity of 0.0012 µmol/min/mg [28]. To identify potential thermostable IspA candidates, enzymes from various biological sources were screened using the BRENDA and UniProtKB databases. This analysis revealed that *G. stearothermophilus*-derived IspA (GsIspA) exhibits a significantly higher specific activity of 4.69 µmol/min/mg [29]. Notably, GsIspA retains full activity after 100 min of treatment at 65 °C [27].

**Fig. 2:**
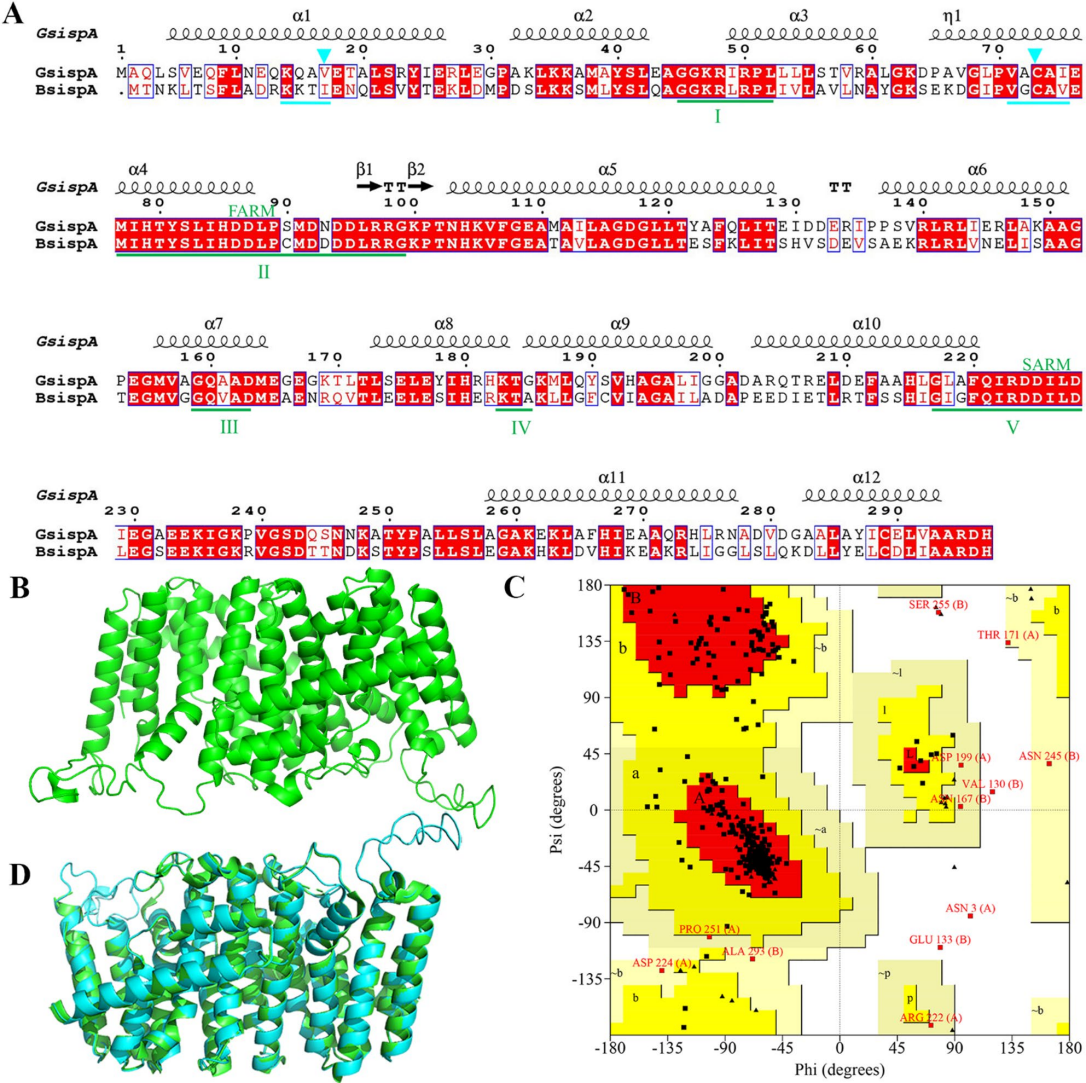
Farnesyl pyrophosphate (FPP) synthase screening and analysis. (A) GsIspA and BsIspA amino acid sequence alignment. Five conserved regions (I–V) are highlighted with green underlining, while semi-conserved regions are marked with blue underlining. Two residues influencing atomic packing, such as Val17 and Cys73 in GsIspA, are denoted by blue inverted triangles. The alignment was created using ESPript [35]. (B) The three-dimensional structure of BsIspA. (C) The Ramachandran plot of the BsIspA structural model. (D) Structural Alignment Done Using PyMol of GsIspA (PDB id: 5AYP) and BsIspA (Modeled) Protein. In the overlapped structure, green represents BsIspA and cyan represents GsIspA

To investigate the molecular basis underlying the superior properties of GsIspA, sequence and structural comparisons were conducted. The amino acid sequence alignment revealed that BsIspA and GsIspA share a sequence similarity of 56.08%, with five conserved regions identified, including the first aspartate-rich motif (FARM, DDX₂-₄D) and the second aspartate-rich motif (SARM, DDXXD) (Fig. 2A). The key aspartate residues chelate Mg²⁺ via their carboxyl groups, thereby positioning the substrates—dimethylallyl diphosphate (DMAPP), geranyl diphosphate (GPP), or isopentenyl diphosphate (IPP)—and catalyzing pyrophosphate group dissociation [9, 30–32]. Additionally, the IspA sequence contains two “semi-conserved” regions, where the side chain volumes of specific amino acids directly influence structural stability by modulating the packing density of the α-helices (Fig. 2A) [27]. Two critical sequence differences between BsIspA and GsIspA may account for their distinct enzymatic activities. The first difference is located at the fifth residue of the FARM functional domain: GsIspA contains serine (Ser⁵), whereas BsIspA contains cysteine (Cys⁵) (Fig. 2A). In GsIspA, the hydroxyl group of serine forms hydrogen bonds that enhance substrate affinity, while its small side chain facilitates the opening of the substrate channel. In contrast, the thiol group of cysteine in BsIspA introduces steric hindrance, potentially interfering with substrate positioning or altering the catalytic microenvironment. The second difference is found within the first “semi-conserved” region, where residue 17 is isoleucine (Ile¹⁷) in BsIspA and valine (Val¹⁷) in GsIspA. Compared to Val, Ile has a bulkier side chain, which increases steric hindrance and tightens the α-helix turn angle. This local conformational contraction subsequently causes a global loosening of the topological structure, ultimately reducing thermal stability [27]. This difference provides a molecular explanation for the superior catalytic efficiency and thermal stability of GsIspA, highlighting precise targets for future enzyme engineering.

The three-dimensional structure of BsIspA was modeled using SWISS-MODEL [33]. Based on GMQE values, the crystal structure of GsIspA (5ayp.pdb) was selected as the template for homology modeling. The resulting BsIspA model demonstrated high confidence (GMQE = 0.78) and displayed a highly α-helical structure, with core helices arranged around a large central cavity (Fig. 2B). The Ramachandran plot obtained from PROCHECK analysis indicated that 90.2% and 7.7% of the residues were in the most favored and allowed regions, respectively. This protein model was reasonable and could be used for subsequent research (Fig. 2C). Structural comparisons between BsIspA and GsIspA showed a high degree of similarity, with a root-mean-square deviation (RMSD) of only 0.213 Å (Fig. 2D). The superior catalytic activity and thermal stability of GsIspA make it particularly well-suited for high-temperature fermentation processes, which are critical for enhancing *B. subtilis* growth and increasing MK-7 production [11, 34]. Given these advantages, GsIspA was identified as a promising candidate for replacing BsIspA in engineered strains aimed at improving MK-7 biosynthesis.

### IspA fusion expression enhances MK-7 production

**Fig. 3:**
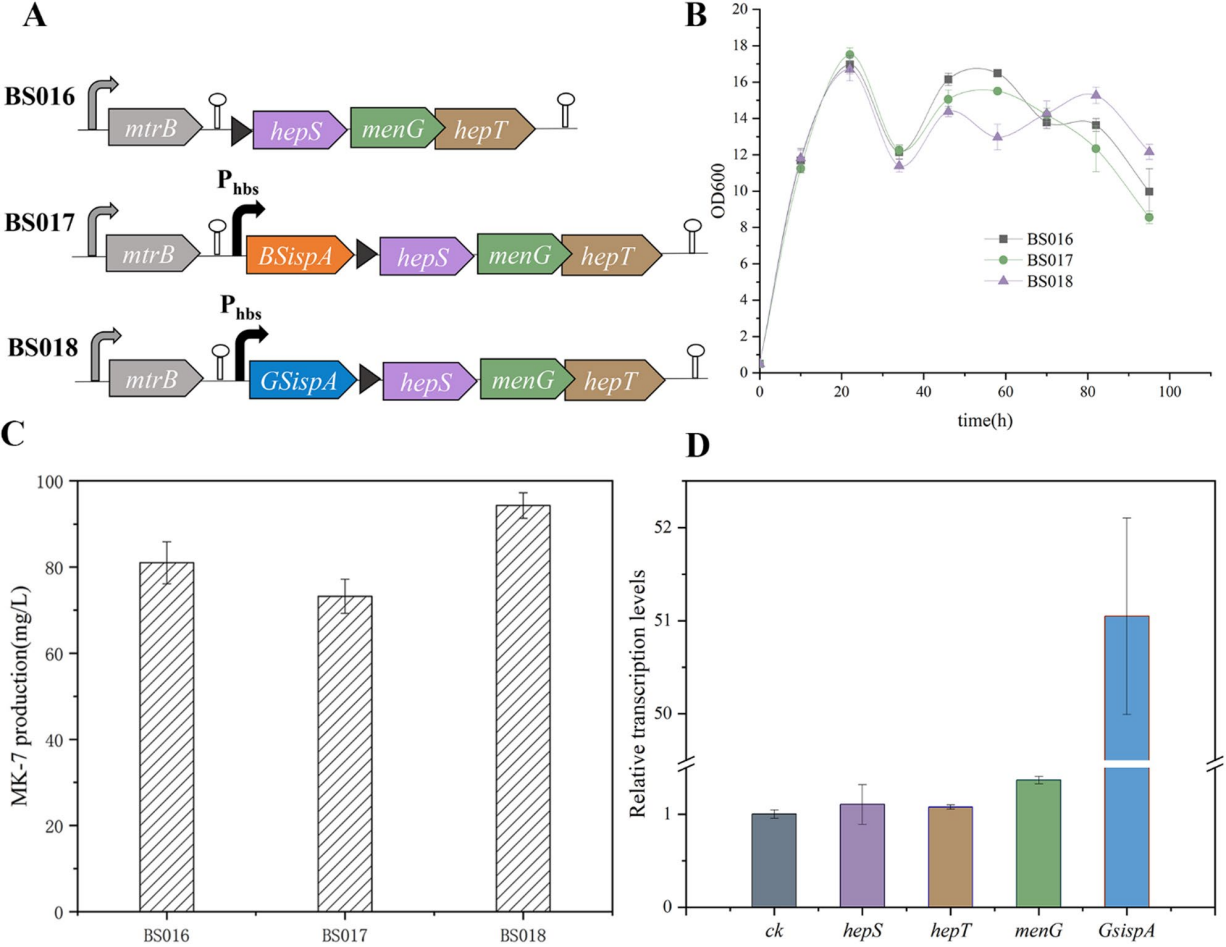
The effects of IspA and HepS/HepT fusion expression on MK-7 production and cell growth. (A) Schematic depicting recombinant strain construction with fusion co-expression of *ispA* and *hepS*/*hepT* from different sources. The black triangle represents 5’UTR of *hepS* (B) Growth curves of the recombinant strains. (C) MK-7 production in recombinant strains after 120 h of fermentation. (D) Relative expression of the modified genes in BS018 compared with that of the corresponding genes in the control strain BS016. The transcript levels of *GsispA*, *hepS*, *menG*, and *hepT* were regarded as 1 in BS016. The results are presented as the mean of three independent biological replicates, with error bars representing the standard deviation

The key enzymes for MK-7 side-chain synthesis, heptaprenyl pyrophosphate synthase (*hepS*/*hepT*), together with demethylmenaquinone methyltransferase (*menG*), constitute the *hepS*-*menG*-*hepT* operon (Fig. 3A). To construct an efficient “metabolic channel”, the Cre/loxP system was employed to modularly integrate *GsispA* into the *hepS*-*menG*-*hepT* operon on the genome of *B.subtilis* BS016. Expression was driven by the strong promoter P_hbs_. The resulting engineered strain was designated BS018 (deposition number: CCTCC NO: M2024004). As a control, the endogenous *BsispA* was integrated and expressed using the same strategy, resulting in BS017 (Fig. 3A).

The fermentation growth curves showed that the three strains (BS016, BS017, and BS018) exhibited similar growth trends during the logarithmic phase; however, BS018 demonstrated a sustained metabolic advantage and the highest biomass during the late stationary phase (Fig. 3B). This might be attributed to the high activity of GsIspA, which ensured sufficient synthesis of FPP. The physical proximity between GsIspA and the downstream enzymes HepS/HepT likely facilitated the rapid capture and utilization of FPP, potentially preventing the accumulation of toxic intermediates (e.g., IPP/DMAPP) and alleviating metabolic stress [18, 36]. Alternatively, the spatial co-localization of enzymes might have formed functional metabolic microcompartments within the cell, significantly shortening the diffusion path of FPP and enabling its more efficient entry into the MK-7 biosynthetic pathway. This coordinated “push-and-pull” mechanism likely enhanced the overall pathway efficiency. In comparison, although BS017 shared the same spatial coupling architecture, the low activity of its endogenous BsIspA became a bottleneck in the system, failing to provide sufficient driving force. The ineffective protein synthesis may have further increased the metabolic burden, leading to the weakest growth and production performance.

The final MK-7 titers in the fermentation broths of the three strains exhibited a clear hierarchical pattern: BS018 achieved an MK-7 yield of 91.1 mg/L (30.4 mg/L/gDCW), representing an 11% improvement over BS016. In contrast, BS017 had the lowest yield of 73.2 mg/L (24.7 mg/L/gDCW) (Fig. 3C). These results confirmed that the strategy of spatial co-localization of the high-activity rate-limiting enzyme GsIspA with the downstream pathway could achieve spatiotemporal coordination of metabolic flux, providing a new paradigm for efficient MK-7 biosynthesis. However, this study’s investigation into the activity differences between GsIspA and BsIspA was primarily based on sequence and structural comparisons. In the future, molecular dynamics simulations could be employed to reveal the dynamic conformational differences between the two enzymes. Combined with site-directed mutagenesis and enzyme kinetics analysis, this approach could more precisely identify key residues affecting catalytic efficiency and stability, providing a theoretical basis for the rational design of improved enzyme components.

To elucidate the molecular basis of the enhanced performance of the engineered strain BS018, qRT-PCR analysis confirmed that under the control of the strong promoter P_hbs_, the expression levels of the *GsispA-hepS-menG-hepT* gene cluster were significantly upregulated compared to the original strain BS016. Specifically, the genes were upregulated by 51.05-, 1.10-, 1.37-, and 1.08-fold, respectively (Fig. 3D). The relatively high expression level of GsispA was due to the absence of this gene in BS016. These results validate the complete transcriptional cascade of the multi-enzyme spatial coupling module and provide a molecular-level explanation for the metabolic advantages observed in the engineered strain.

### BS018 batch fermentation

**Fig. 4:**
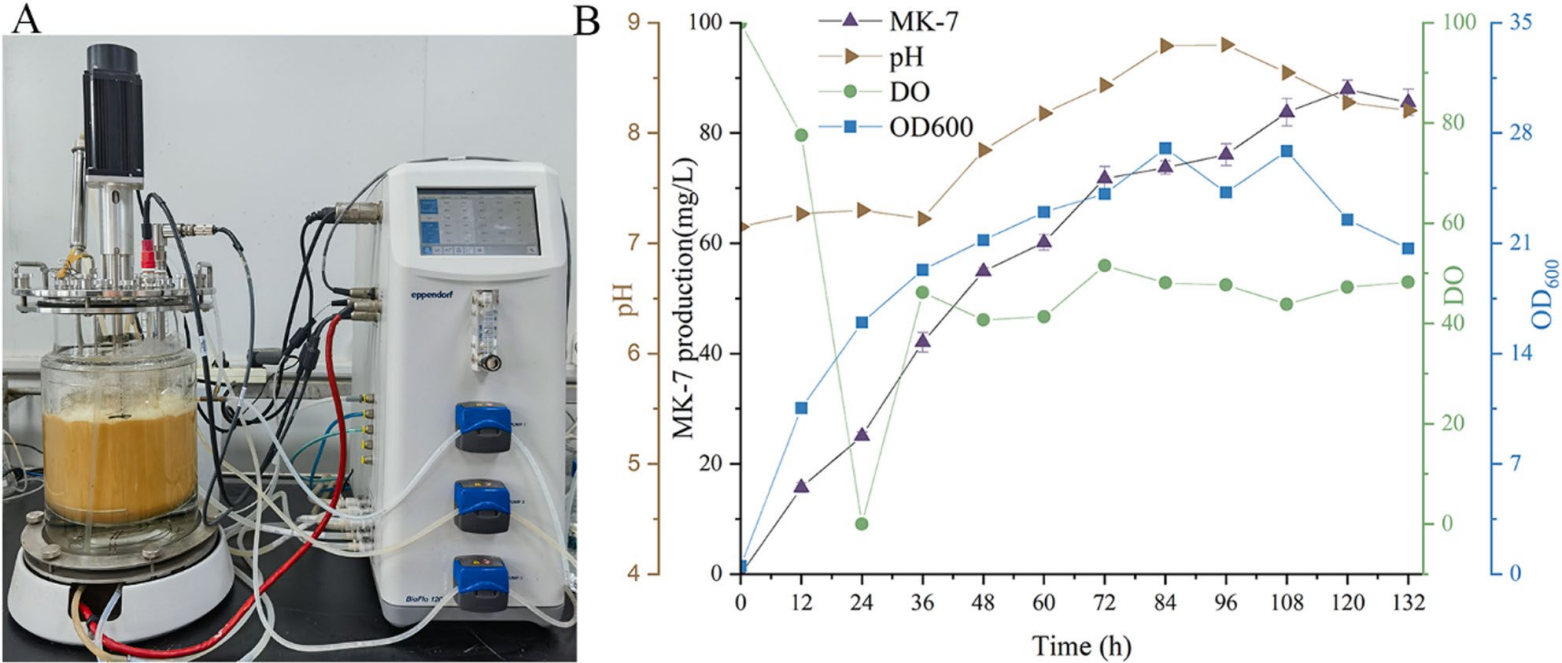
BS018 batch fermentation in a 5-L fermenter. (A) Schematic diagram of the 5-L fermenter setup. (B) Fermentation process curves of BS018 in the 5 L fed-batch. The production of MK-7 was determined in three technical replicates

To evaluate the production performance of BS018 under scaled-up cultivation conditions, a validation experiment was conducted in a 5-L fermenter at 40 °C for 120 h (Fig. 4A). The growth curve showed that BS018 entered the stationary phase 48 h after fermentation; a declining phase was observed from 108 to 120 h. The DO curve revealed that during 0–24 h of fermentation, DO levels dropped sharply, reaching the lowest point (0.5%) at 24 h. This indicated that BS018 was in a rapid logarithmic growth phase, with a significant increase in oxygen demand. After 36 h, DO levels began to recover and remained stable. Combined with growth curve analysis, this recovery in DO levels may be attributed to a decrease in the number of viable cells and a reduction in their metabolic activity. The pH profile showed that during 0–36 h of fermentation, the pH of the fermentation broth remained stable at approximately 7.2. After 36 h, the pH began to rise steadily, reaching 8.18 at 60 h (Fig. 4B). Based on the combined analysis of the growth and DO curves, this pH increase was likely due to carbon source depletion, which triggered a metabolic shift in the cells [37]. Specifically, the cells might have initiated the deamination of nitrogen sources, producing α-ketoglutarate, which enters the TCA cycle to sustain basic metabolic activity. However, alkaline byproduct accumulation during this process directly causes the pH of the fermentation broth to increase, coinciding with the onset of the cell decline phase. MK-7 production curve showed a continuous upward trend from 0 to 36 h, reaching its peak at 120 h, with a maximum yield of 87.9 mg/L (Fig. 4B). Compared with that in shake-flask fermentation, the MK-7 yield in the 5-L fermenter was slightly lower. This discrepancy might be attributed to excessive cell proliferation during the early stages of fermentation, which led to rapid carbon source depletion. At 36 h, the carbon source was nearly exhausted, significantly limiting cell MK-7 overproduction. In the future, MK-7 yield will be improved by reducing the inoculum dose to slow the initial specific growth rate or implementing fed-batch fermentation from 36 h to supplement the carbon source.

### Optimization of fermentation parameters for BS018 in shake-flask culture

Optimizing fermentation parameters is a critical step in enhancing target product synthesis [8]. In this study, the fermentation conditions were optimized using MK-7 production in BS018 as an indicator. Three factors were considered in the single-factor shake-flask experiments, i.e., liquid loading, initial pH, and inoculum dose.

#### Liquid loading optimization

**Fig. 5:**
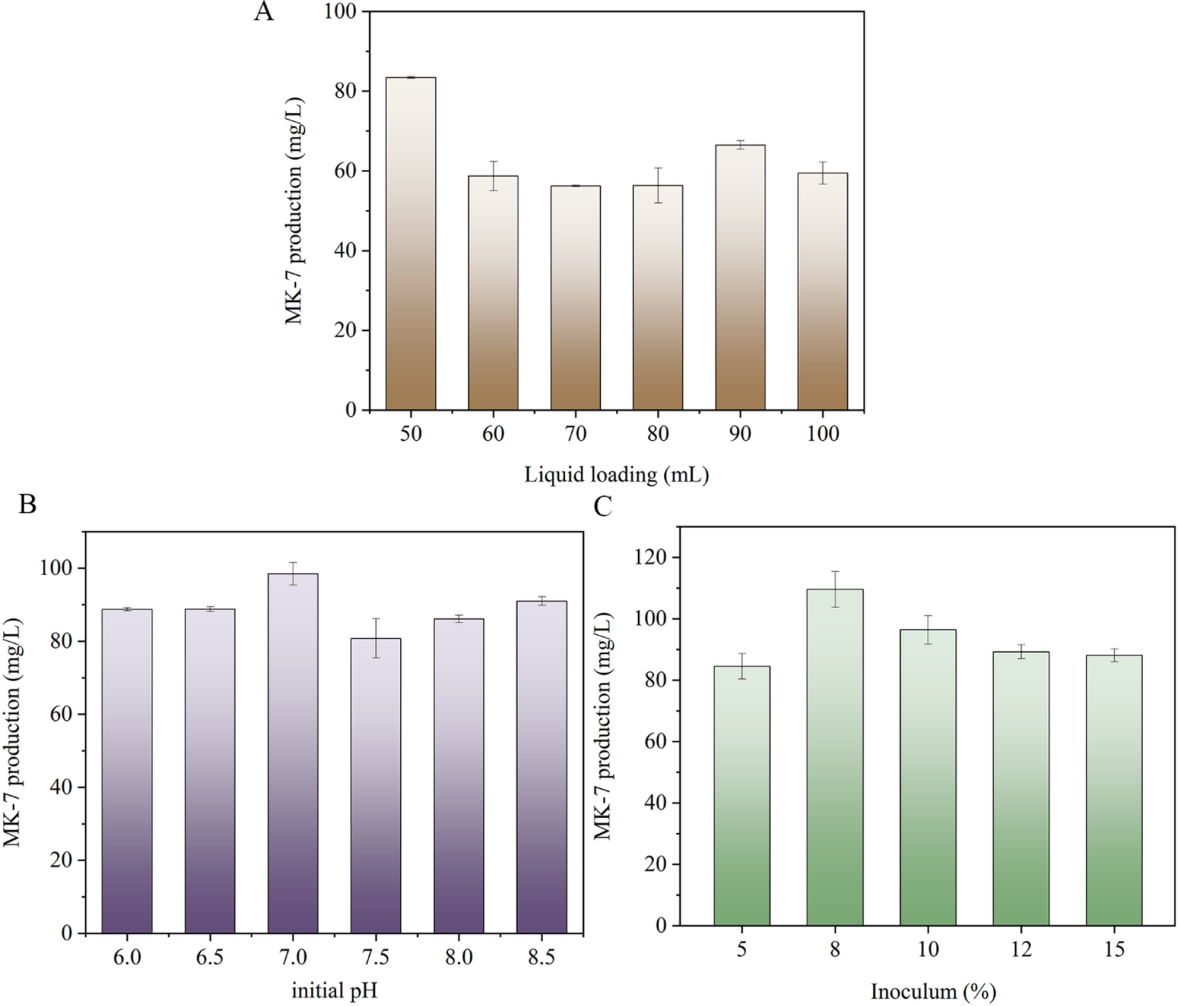
Effects of liquid loading (A), initial pH (B), inoculum dose (C) on MK-7 production by strain BS018 in shake-flask culture. The results are presented as the mean of three independent biological replicates, with error bars representing the standard deviation

In *B. subtilis*, an aerobic bacterium, oxygen supply is a key factor that influences MK-7 synthesis [38]. Oxygen not only regulates the expression of MK-7 metabolism-related genes, but also significantly improves glycerol utilization, reduces the expression of sporulation-related genes, and strengthens the bacterial antioxidant defense system [3, 38, 39]. In shake-flask cultures, excessive liquid loading reduces the contact area between the medium and air, lowering the DO levels, which leads to byproduct accumulation, including lactic acid, acetic acid, and ethanol, all of which inhibit cell growth and target metabolite synthesis [40]. Conversely, insufficient liquid loading might result in inadequate nutrient supply, causing cells to prematurely enter the decline phase and reducing fermentation efficiency. In this study, 500 mL shake flasks were used as fermentation vessels. Furthermore, different liquid loadings (50, 60, 70, 80, 90, and 100 mL) of fermentation medium were tested. The other fermentation conditions included seed culture inoculum dose of 5%, initial pH of 7.3, and cultivation at 40 °C with shaking (250 rpm) for 120 h. MK-7 yield in BS018 reached its highest value (83.4 mg/L), when the liquid loading was 50 mL, which was significantly higher than that in the other groups (Fig. 5A). As the liquid loading increased, the yield of MK-7 gradually decreased. Therefore, a liquid loading of 50 mL/500 mL was determined to be optimal for MK-7 production in BS018.

#### Initial pH optimization

Among the various environmental factors affecting fermentation, pH is of critical importance because microbial activity, enzyme stability, membrane permeability, and substrate toxicity are pH-dependent [41]. In addition, pH fluctuations during fermentation inevitably result in alterations in metabolic function [41]. In this study, the initial pH was set at 6.0, 6.5, 7.0, 7.5, 8.0, and 8.5. Other fermentation conditions were, a liquid loading of 50 mL/500 mL, a seed culture inoculum dose of 5%, and cultivation at 40 °C with shaking (250 rpm) for 120 h. The experimental results showed that the highest MK-7 yield in BS018 was 98.5 mg/L at an initial pH of 7.0. In contrast, MK-7 production significantly decreased under excessively high (≥ 8.0) or low (≤ 6.5) pH conditions (Fig. 5B). These findings are consistent with the those of Chen et al. [34] in their fermentation optimization study of the MK-7-producing strain BS20DFHG, further confirming the critical regulatory role of the initial pH in MK-7 synthesis.

#### Inoculum optimization

Seed culture inoculum could influence microbial growth dynamics and metabolic activity. An excessive inoculum dose could lead to rapid bacterial growth, resulting in insufficient DO and premature nutrient depletion, which causes the cells to enter the decline phase early and inhibit the synthesis of the target product. Conversely, an insufficient inoculum dose might result in a low initial cell concentration, a prolonged lag phase, an extended fermentation cycle, and reduced production efficiency [42]. In this study, the inoculum doses were 5%, 8%, 10%, 12%, and 15%. Other fermentation conditions were, a liquid loading of 50 mL/500 mL, an initial pH of 7.0, and cultivation at 40 °C with shaking (250 rpm) for 120 h. When the inoculum dose was 8%, the MK-7 yield in BS018 reached the highest value of 109.6 mg/L (Fig. 5C). This indicates that under these conditions, the initial cell concentration and supply of oxygen and nutrients in the medium were well balanced. The cells smoothly entered the exponential growth phase and maintained a high metabolic activity for an extended period, fully activating the MK-7 synthesis pathway and achieving higher yields. This study demonstrated that an inoculum dose of 8% was optimal for BS018 MK-7 production.

## Conclusions

This work demonstrates that the directed screening of superior heterologous enzymes, combined with the spatial co-assembly of sequential enzymes into a functional complex, constitutes an effective strategy for alleviating rate-limiting steps in complex metabolic pathways. By reconstructing a localized “metabolic channel” for FPP utilization, we effectively enhanced the flux towards MK-7 biosynthesis in *B. subtilis*. This spatial co-assembly strategy not only successfully addressed the specific bottleneck in MK-7 synthesis, leading to a yield of 109.6 mg/L, but also establishes a generalizable metabolic engineering paradigm that can be extrapolated to optimize the biosynthesis of other valuable terpenoids and natural products with complex branching pathways.

## Supplementary Information

Below is the link to the electronic supplementary material.

Supplementary Material 1

## Data Availability

All data generated or analysed during this study are included in this published article and its supplementary information files.

## Enzymes

MenF: Isochorismate synthase
MenA: 1,4-dihydroxy-2-naphthoate heptaprenyltransferase
MenG: Demethylmenaquinone methyltransferase
IspA: Farnesyl diphosphate synthase
HepS/HepT: Heptaprenyl diphosphate synthase component I/II
DXS: 1-deoxyxylulose-5-phosphate synthase
Fni: Isopentenyl-diphosphate delta-isomerase
AroA: 3-Deoxy-7-phosphoheptulonate synthase
UPPs: Undecaprenyl diphosphate synthase

## Metabolites

Gly: Glycerol
G3P: Glyceraldehyde-3-phosphate
PYR: Pyruvate
E4P: Erythrose 4-phosphate
DAHP: 3-deoxy-arabino-heptulonate 7-phosphate
CHA: Chorismite
DXP: 1-deoxyxylulose-5-phosphate
MEP: Methyl-erythritol-4-diphosphate
HMBPP: 1-hydroxy-2-methyl-2-butenyl 4-diphosphate
DMAPP: Dimethylallyl diphosphate
IPP: Isopentenyl diphosphate
FPP: Farnesyl diphosphate
HepPP: Heptaprenyl diphosphate
ICHA: Isochorismate
DHNA: 1,4-dihydroxy-2-naphthoate
DMK-7: 2-demethylmenaquinone-7
MK-7: Menaquinone-7
MEP: 2-C-methyl-d-erythritol 4-phosphate
DMK-7: Demethylmenaquinone-7
