# Supplementary material for: Directed screening and spatial coupling of farnesyl diphosphate synthase for enhancing menaquinone-7 production in Bacillus subtilis
Source: Microb Cell Fact. 2026 Jan 14;25:44. doi: 10.1186/s12934-026-02930-1 (PMC12903253; doi:10.1186/s12934-026-02930-1)
Supplement: Supplementary file 1 — Supplementary Material 1 [file 12934_2026_2930_MOESM1_ESM.docx]

Supplementary materials for

Directed screening and spatial coupling of farnesyl diphosphate synthase to improve Menaquinone-7 production in *Bacillus subtilis*

XiuminDing^1,2†^, Rui Zhang^2,3†^, Ying Liu^1^, Liang Hong^1^, Yalan Feng^4^, Qiang Li^4*^, Zhiming Zheng^2*^, Genhai Zhao^2*^

^1^School of Laboratory Medicine, Wannan Medical College, Wuhu 241002, China;

^2^Institute of Intelligent Machines, Hefei Institutes of Physical Science, Chinese Academy of Sciences, Hefei 230031, China;
^3^ School of Life Sciences, Anhui Medical University, Hefei, 230012, China

^4^ School of Life Sciences, Wuchang University of Technology, Wuhan 430223, China

*Corresponding author: Qiang Li, Zhiming Zheng and Genhai Zhao

†Rui Zhang: the same contribution to the paper
Tel/Fax: +86-0553-3932417,

e-mail: [liqiang190901@163.com](mailto:liqiang190901@163.com); zhengzhiming2014@163.com; [zhgh327@yeah.net](mailto:zhgh327@yeah.net)

Table S1. Strains and plasmids used in this study

| Strain | characteristics | reference |
| --- | --- | --- |
| BS016 | BS168, P_43_-*dxs*, P_43_-*fni*, P_43_-*dxr*, P_43_-*menF*, P_hbs_-*aroA*, Δ*dhbB*:: P_43_-*menA*, Δ*amyE*:: P_43_-*pos5P*, P_43_-*sodA*, P_43_-*katE* | Lab stock |
| BS017 | BS168, P_43_-*dxs*, P_43_-*fni*, P_43_-*dxr*, P_43_-*menF*, P_hbs_-*aroA*, Δ*dhbB*:: P_43_-*menA*, Δ*amyE*:: P_43_-*pos5P*, P_43_-*sodA*, P_43_-*katE*, P_hbs_-*GSispA* | This work |
| BS018 | BS168, P_43_-*dxs*, P_43_-*fni*, P_43_-*dxr*, P_43_-*menF*, P_hbs_-*aroA*, Δ*dhbB*:: P_43_-*menA*, Δ*amyE*:: P_43_-*pos5P*, P_43_-*sodA*, P_43_-*katE*, P_hbs_-*BSispA* | This work |
| Plasmids |  |  |
| p7C6P_hbs_ | pMD18-T ligated with lox71-cm-lox66 cassette and P_hbs_ promoter | Lab stock |
| PDGC | Amp, Km, *E. coli*−*B. subtilis* shuttle vector, containing *cre* under the control of P_spac_ | Lab stock |

Table S2. Primers used in this study

| Primers | Sequences（5＇→3＇） |
| --- | --- |
| bsispa2-R | GCTCGTTAATTTATTTGTCATGTGTACATTCCTCTCTTACCCCA |
| bsispa3-F | GGGGTAAGAGAGGAATGTACACATGACAAATAAATTAACGAGC |
| bsispa3-R | CGCTTACGCAGGTATCATTTTAGTGATCTCTTGCCGCAATT |
| bsispa4-F | TTGCGGCAAGAGATCACTAAAATGATACCTGCGTAAGCGAAT |
| gsispa2-R | TCAACTGAAAGCTGCGCCATGTGTACATTCCTCTCTTACC |
| gsispa3-F | GTAAGAGAGGAATGTACACATGGCGCAGCTTTCAGTTGAAC |
| gsispa3-R | GCTTACGCAGGTATCATTTTAATGGTCGCGGGCGGCGACCAGTC |
| gsispa4-F | TCGCCGCCCGCGACCATTAAAATGATACCTGCGTAAGCGAAT |
| heps1-F | GGGCATATGTGCTAGAATCGAAATT |
| heps1-R | TCCCCGGGTACCGAGCTCGATAGCATAAAAAAAAGAAATT |
| heps2-F | ATTTCTTTTTTTTATGCTATCGAGCTCGGTACCCGGGGAT |
| heps4-R | TGCGCCTTCCTTTACATTCATGAT |
| **qRT-PCR** |  |
| hept-qpcr.F | GTACGTTCCGATTACCCGCTT |
| hept-qpcr.R | CGATTGTCCCACTTTGCTTTG |
| hepS-qpcr.F | GATTATTTCAGCGGGCTGTACTAC |
| hepS-qpcr.R | GATTCAACGATGCCGACACTTTCG |
| menG-qpcr.F | GTCGGCGAGCAGAAAGTAAAAGA |
| menG-qpcr.R | AAGCCAATGGTGACATAATC |
| GSispA-qpcr.F | CAAAAACAGGCGGTGGAAACAGC |
| GSispA-qpcr.R | TCATCATGGATCAAAGAGTACG |

Table S3： sequence used in this study

| Gene | sequence |
| --- | --- |
| *GSispA* | ATGGCGCAGCTTTCAGTTGAACAGTTTCTCAACGAGCAAAAACAGGCGGTGGAAACAGCGCTCTCCCGTTATATAGAGCGCTTAGAAGGGCCGGCGAAGCTGAAAAAGGCGATGGCGTACTCATTGGAGGCCGGCGGCAAACGAATCCGTCCGTTGCTGCTTCTGTCCACCGTTCGGGCGCTCGGCAAAGACCCGGCGGTCGGATTGCCCGTCGCCTGCGCGATTGAAATGATCCATACGTACTCTTTGATCCATGATGATTTGCCGAGCATGGACAACGATGATTTGCGGCGCGGCAAGCCGACGAACCATAAAGTGTTCGGCGAGGCGATGGCCATCTTGGCGGGGGACGGGTTGTTGACGTACGCGTTTCAATTGATCACCGAAATCGACGATGAGCGCATCCCTCCTTCCGTCCGGCTTCGGCTCATCGAACGGCTGGCGAAAGCGGCCGGTCCGGAAGGGATGGTCGCCGGTCAGGCAGCCGATATGGAAGGAGAGGGGAAAACGCTGACGCTTTCGGAGCTCGAATACATTCATCGGCATAAAACCGGGAAAATGCTGCAATACAGCGTGCACGCCGGCGCCTTGATCGGCGGCGCTGATGCCCGGCAAACGCGGGAGCTTGACGAATTCGCCGCCCATCTAGGCCTTGCCTTTCAAATTCGCGATGATATTCTCGATATTGAAGGGGCAGAAGAAAAAATCGGCAAGCCGGTCGGCAGCGACCAAAGCAACAACAAAGCGACGTATCCAGCGTTGCTGTCGCTTGCCGGCGCGAAGGAAAAGTTGGCGTTCCATATCGAGGCGGCGCAGCGCCATTTACGGAACGCCGACGTTGACGGCGCCGCGCTCGCCTATATTTGCGAACTGGTCGCCGCCCGCGACCATTAA |
| BSispA | ATGACAAATAAATTAACGAGCTTTCTGGCGGACCGGAAAAAAACAATTGAAAATCAGCTTTCTGTCTATACAGAAAAGCTTGATATGCCGGACTCATTAAAGAAATCTATGCTATATTCTCTACAGGCCGGCGGAAAGCGGTTGCGGCCTCTGATTGTACTGGCTGTTTTAAATGCATATGGAAAAAGCGAAAAAGACGGCATTCCGGTGGGCTGTGCTGTCGAAATGATTCACACGTATTCGTTAATTCATGATGATCTTCCATGCATGGATGATGACGATTTGCGCCGCGGGAAGCCGACAAACCATAAAGTGTTTGGTGAAGCGACGGCAGTATTAGCGGGTGACGGGCTGCTCACAGAAAGCTTTAAGCTGATTACCTCCCACGTGTCAGACGAGGTGTCAGCAGAAAAGCGCCTGCGGCTTGTGAATGAACTGATTTCAGCGGCAGGCACCGAAGGCATGGTCGGTGGGCAAGTAGCTGATATGGAAGCGGAAAACCGACAAGTCACGCTTGAAGAGCTCGAATCCATTCATGAACGGAAAACTGCCAAGCTCCTTGGCTTTTGTGTAATCGCCGGTGCTATTTTGGCTGATGCGCCTGAGGAAGACATTGAAACACTGCGTACCTTCAGCAGCCACATTGGAATCGGATTTCAAATCAGAGACGATATTTTAGATTTAGAAGGCAGTGAAGAGAAAATCGGCAAACGTGTCGGCTCGGATACCACAAATGACAAATCGACATACCCGTCGCTTCTTTCATTGGAAGGGGCCAAACATAAATTGGATGTTCATATAAAAGAGGCGAAGCGATTGATCGGCGGACTCTCTCTTCAAAAAGACCTTTTATATGAGCTTTGTGATTTAATTGCGGCAAGAGATCACTAA |

Note S1. Abbreviations

Enzymes: MenF, isochorismate synthase; MenA, 1,4-dihydroxy-2-naphthoate heptaprenyltransferase; MenG, demethylmenaquinone methyltransferase; IspA, farnesyl diphosphate synthase; HepS/HepT, heptaprenyl diphosphate synthase component I/II; DXS, 1-deoxyxylulose-5-phosphate synthase; Fni, isopentenyl-diphosphate delta-isomerase; AroA, 3-Deoxy-7-phosphoheptulonate synthase; undecaprenyl diphosphate synthase, UPPs;

Metabolites: Gly, Glycerol; G3P, glyceraldehyde-3-phosphate; PYR, pyruvate; E4P, erythrose 4-phosphate; DAHP, 3-deoxy-arabino-heptulonate 7-phosphate; CHA, Chorismite; DXP, 1-deoxyxylulose-5-phosphate; MEP, methyl-erythritol-4-diphosphate; HMBPP, 1-hydroxy-2-methyl-2-butenyl 4-diphosphate; DMAPP, dimethylallyl diphosphate; IPP, isopentenyl diphosphate; FPP, farnesyl diphosphate; HepPP, heptaprenyl diphosphate; ICHA, isochorismate; DHNA, 1,4-dihydroxy-2-naphthoate; DMK-7, 2-demethylmenaquinone-7; MK-7, menaquinone-7; MEP: 2-C-methyl-d-erythritol 4-phosphate;
